# Diagnostic delay length and disease severity are the main predictors of sleep apnea in acromegaly patients

**DOI:** 10.3389/fendo.2026.1928818

**Published:** 2026-10-12

**Authors:** Rosa Pirchio, Renata Simona Auriemma, Stefano Sanduzzi Zamparelli, Paola Bonavolontà, Claudia Merola, Angelo Canora, Claudia Pivonello, Alessandro Sanduzzi Zamparelli, Rosario Pivonello, Annamaria Colao

**Affiliations:** 1 Dipartimento di Medicina Clinica e Chirurgia, Sezione di Endocrinologia, Diabetologia, Andrologia e Nutrizione, Università Federico II di Napoli, Naples, Italy; 2 Division of Pneumology and Semi‑Intensive Respiratory Therapy, A. Cardarelli Hospital, Naples, Italy; 3 Maxillofacial Surgery Unit, Department of Neurosciences, Reproductive and Odontostomatological Sciences, University of Naples Federico II, Naples, Italy; 4 Istituti Clinici Scientifici Maugeri IRCCS, Pulmonary Rehabilitation Unit of Telese Terme Institute, Telese Terme, Italy; 5 Dipartimento di Sanità Pubblica, Università Federico II di Napoli, Naples, Italy; 6 Department of Clinical Medicine and Surgery, University of Naples “Federico II”, Naples, Italy; 7 UNESCO Chair for Health Education and Sustainable Development, University of Naples “Federico II”, Naples, Italy; 8 ERN Lung, Frankfurt am Main, Germany

**Keywords:** acromegaly, diagnostic delay, IGF-I, OSA, saturation, sleep apnea

## Abstract

**Context:**

Obstructive sleep apnea (OSA) affects up to 80% of patients at diagnosis of acromegaly and often persists despite treatment. Currently, patients at risk for OSA are recommended to be identified to offer polygraphy evaluation.

**Purpose:**

To study the prevalence and risk factors for OSA in patients with acromegaly.

**Methods:**

Prospective, single-center study; polygraphy was performed on 36 patients with acromegaly (age 54.1 ± 12.9 years).

**Results:**

OSA was found in 17 patients (47.2%), mostly mild (64.7%). Patients with OSA were older both at diagnosis (p = 0.02) and at polygraphy (p = 0.008), had a longer diagnostic delay (p = 0.003) and higher BMI (p = 0.03). Patients with uncontrolled acromegaly exhibited a significantly higher number of apneas (p=0.02) and obstructive apneas (p=0.04) and had significantly lower minimum oxygen saturations (p=0.02) and a higher percentage of time spent with oxygen saturation <90% (p=0.03) as compared to those with full disease control. Diagnostic delay was the best predictor of apnea-ipopnea index (AHI, p=0.04) and oxygen desaturation index (ODI, p=0.04) scores, independently on BMI. Insulin resistance (p = 0.05), glucose profile impairment (p = 0.05) and STOP-Bang score (p=0.001), but not Epworth Sleepiness Scale score (p = 0.96), were significantly higher in patients with OSA as compared to those without.

**Conclusion:**

Diagnostic delay and patients’ age, rather than disease status, appear to predict the development of OSA and influence its severity. These findings reinforce the importance of early identification of patients at higher risk for OSA.

## Introduction

Besides growth hormone (GH) and Insulin-like growth factor 1 (IGF-I) excess (1), acromegaly results in an increased mortality due to its strong association with systemic complications (2, 3). Hormonal imbalance also results in the occurrence of respiratory complications, whose exact prevalence and impact on overall mortality is yet to be fully elucidated in acromegaly patients.

Acromegaly-induced impairments of the respiratory system mainly include sleep apnea and respiratory insufficiency (3). Acromegaly patients are mostly affected by the obstructive sleep apnea (OSA) rather than the central sleep apnea (4). OSA is very common among acromegaly patients, raising its prevalence up to 80% at acromegaly diagnosis (5). Particularly, acromegaly patients experience anatomical modifications affecting craniofacial bones and soft tissues (swelling of the tongue, changes in the respiratory mucosa and cartilages, lung volume, and rib cage geometry), and alterations in muscle and lung structure, which, together with the direct negative effect of GH and IGF-I excess on brain control of respiration, lead to respiratory dysfunction (3). Particularly, soft palate has been reported to be elongated and thickened and the vallecula-to-tongue distance to be increased in acromegaly patients with OSA as compared to those without (6); a thicker lateral pharyngeal wall and decreased maximal transverse diameter and cross-sectional area of the airway have been also described in acromegaly (4). The Apnea-Hypopnea Index (AHI), a key parameter widely used to measure the severity of OSA in clinical settings, has been found correlated to vallecula-to-tongue distance (6), tongue base hypertrophy (7), thickness of the soft palate and lateral pharyngeal (4), and inversely with maximal transverse diameter and cross-sectional area of the airway in the soft palate plane (4) in acromegaly patients.

According to available evidence, independent predictors of OSA in acromegaly include disease activity, older age, and greater neck circumference (2), whereas no clear role of biochemical control of acromegaly on OSA prevalence and severity has been demonstrated so far.

Current international guidelines about the management of acromegaly complications (5) recommend to assess OSA at diagnosis of acromegaly, particularly in those individual subjects reporting symptoms suggestive of OSA, such as snoring, pauses in breathing, waking up feeling choked, and excessive sleepiness in daytime, and suggest to use a sleep questionnaire, such as the Epworth sleepiness scale (ESS), to evaluate the potential risk of OSA (5). If OSA is suspected, polygraphy, the gold standard for OSA diagnosis, should be performed to confirm OSA diagnosis and carry out the treatment (5).

However, very recently a survey evaluating the risk of OSA using the sleep questionnaires in more than 200 acromegaly patients has shown that 19.3% were at risk of daily sleepiness according to ESS, thus leading to the hypothesis that a clinical investigation of OSA risk based on questionnaires might underestimate the prevalence of respiratory dysfunctions (8). Conversely, a higher OSA prevalence of approximately 70.9% (range 43.7-97%) has been reported in observational real-world studies using polygraphy to assess OSA presence and severity in acromegaly, as shown in **Table 1** (4, 9–14). Identifying OSA is clinically relevant, since OSA could contribute to the increased mortality in acromegaly. Indeed, besides the worsening of QoL due to OSA symptom, OSA has a well-known negative impact on the cardiovascular system (15), being OSA an independent risk factor for ischemic heart disease, arrhythmia, and cardiomyopathy in acromegaly patients already at higher cardiovascular risk due to GH and IGF-I excess *per se* (5).

**Table 1:** Summary of OSA prevalence in polygraphy-based studies.

Study First author, year (ref)	Country	Population	Number of patients	OSA
*Gordillo, 2012* (11)	Mexico	Controlled and uncontrolled patients	35	34 (97.0%)
*Annamalai, 2013* (14)	United Kingdom	Newly diagnosed patients	29	23 (79.3%)
*Chemla, 2014* (13)	France	Newly diagnosed patients	16	7 (43.7%)
*Turan, 2016* (6)	Turkey	Patients with active acromegaly and in remission	27	17 (62.9%)
*Guo, 2018* (4)	China	Newly diagnosed patients	25	13 (52.0%)
*Wolters, 2020* (12)	The Netherlands	Newly diagnosed patients	27	20 (74.1%)
*Celik, 2021* (9)	Turkey	Patients with active acromegaly and symptoms suggestive of sleep disorders	32	25 (78.1%)
*Powlson, 2023* (10)	United Kingdom	Newly diagnosed patients	39	31 (79.0%)
*Pilarska, 2025* (7)	Poland	Newly diagnosed patients	30	23 (76.7%)
Pirchio, 2026**	Italy	Controlled and uncontrolled patients	36	17 (47.2%)
*Total*			296	210 (70.9%)

Further, OSA has been demonstrated to exert a negative impact on gluco-insulinemic metabolism, as in the general population OSA has been suggested to promote its impairment *via* the effects of sleep fragmentation, sympathetic excitation and intermittent hypoxia on pancreatic β-cell function, insulin sensitivity, and systemic inflammation (16). Similarly to cardiovascular health, the impairment in glucose metabolism is frequently reported in acromegaly patients, resulting in a diabetes mellitus (DM) prevalence of approximately 30% (17).

Overall, biochemical control of acromegaly is reported to result in the improvement of OSA severity, although OSA may persist despite proper acromegaly treatment (5, 13, 14, 18). These findings were confirmed by a recent meta-analysis (19), showing that, albeit OSA prevalence was not significantly different between patients with active and controlled disease, a significant decrease of AHI was found after both medical or surgical treatment for acromegaly, thus implying that the reduction in GH and IGF-I levels might contribute to the improvement of respiratory function in acromegaly.

Based on this evidence, one could speculate that OSA assessment should be routinely offered to all patients with acromegaly to early identify those with a greater respiratory and cardiometabolic risk. To date, no study has specifically investigated polygraphy findings in acromegaly patients, performed systemically regardless of symptoms suggestive for a potential risk of OSA, assessed with questionnaires. Given these gaps, this study aimed to better describe the prevalence and the faces of OSA in acromegaly patients, investigating the role of anthropometric, metabolic, and hormonal parameters as risk factors for OSA in patients with acromegaly by a nocturnal cardio-respiratory polygraphy.

## 2 Patients and methods

### 2.1 Study design

This prospective cross-sectional study included patients with a well-established diagnosis of acromegaly according to the international guidelines (20–22) at the Neuroendocrine Disease Unit of “Federico II” University of Naples who consecutively underwent a complete polygraphy evaluation from November 2023 to May 2025.

Inclusion criteria were: 1) age 18–80 years; 2) confirmed diagnosis of acromegaly, 3) patients naïve to any treatment (at diagnosis), or 4) medically/surgically treated at the time of evaluation.

The exclusion criteria were represented by the presence of the following conditions: 1) diagnosis of OSA already known; 2) refuse to perform polygraphy; 3) patients without a complete lung evaluation; 4) unavailability of complete clinical and/or hormonal data.

To find a significant difference between patients with and without OSA by large effect size (Cohen’s d = 0.8), a sample size of 25 achieved 90% power to reject the null hypothesis of equal means with a significance level (alpha) of 0.05. With an expected dropout rate of 20%, the sample size has been increased to 30 patients to be enrolled.

This research complied with the Declaration of Helsinki. All patients provided written, informed consent to approve polygraphy diagnosis testing, according to the Italian privacy policy. The study was approved by the local Ethic Committee (259/2023).

### 2.2 Study population

From November 2023 to May 2025, 41 consecutive acromegaly patients performed polygraphy and were considered potentially eligible as they fulfilled the inclusion criteria of the study. Among them, 2 patients were excluded due to incomplete clinical data and 3 due to incomplete polygraphy recording. Therefore, data of thirty-six patients with acromegaly (14 men and 22 women, aged 54.1 ± 12.9 years) were analyzed in the current study.

Specifically, 29 patients (80.5%) harbored a macroadenoma at acromegaly diagnosis, whereas pituitary MRI revealed a microadenoma in 7 patients (19.5%). Among them, 5 patients (13.9%) were treatment-naïve and 32 (86.1%) were receiving or had received treatment for acromegaly, including 21 (58.3%) treated both with surgery and medical therapy, and 10 cured after neurosurgery (27.8%). Age at acromegaly diagnosis was 46.9 ± 11.8 years and acromegaly diagnostic delay was 7.2 ± 4.9 years. The presumed onset of acromegaly and, consequently, the diagnostic delay before the diagnosis of acromegaly were assessed by comparing photographs taken over a period of 10–30 years and by interviewing the patients as to the date of the occurrence of acral enlargement and facial disfigurement.

According to Katznelson et al. (21), acromegaly was considered fully controlled in the presence of sex- and age-related normal IGF-I levels, expressed as an IGF-I x Upper limit of normal (ULN) <1.0 (19 patients). Patients with IGF-I 1.1-1.3 x ULN were considered partly controlled (IGF-I 1.1-1.3 x ULN, 9 patients), whereas patients were defined uncontrolled if IGF-I was > 1.3 x ULN (8 patients).

### 2.3 Study protocol

In this cross-sectional study, all patients underwent nocturnal cardiorespiratory polygraphy in ambient air, performed by trained sleep physicians with more than 5 years of experience in accordance with AASM recommendations for diagnostic testing in adult OSA (23). Overnight sleep monitoring was realized with a level III portable diagnostic device (Sleep&Go^®^, Sibelmed^®^, Barcelona, Spain). Measurements included chest wall and abdominal movements, nasal airflow, oxygen saturation (SpO2), heart rate (HR), and body position. Respiratory data included the number of events: obstructive apneas, obstructive hypopneas, central apneas, and mixed apneas, scored according to the American Academy of Sleep Medicine guidelines (23).

Oxygen desaturation was defined as a ≥3% decrease in SpO_2_ from baseline. The percentage of recording time spent with oxygen saturation below 90% (T90) was also calculated. OSA degree of severity was defined according to the apnea-hypopnea index (AHI), calculated as the total number of apneas and hypopneas per hour of recording time (23). An AHI <5 events/hour was considered within the normal range. OSA severity was classified according to the standard criteria as mild (AHI 5–14.9 events/hour), moderate (AHI 15–29.9 events/hour), and severe (AHI ≥30 events/hour) (23).

In these patients, the oxygen desaturation index (ODI), defined as the number of ≥3% oxygen desaturation events per hour of recording time, was also recorded. Nocturnal hypoxemia was defined as a T90 ≥ 30% or the presence of at least one ≥5-minute period with SpO_2_ ≤ 90%, reaching a nadir of ≤85% during the recording (23).

Positional obstructive sleep apnea (pOSA) was defined as a supine AHI at least twice the non-supine AHI (24).

The following patients’ data at polygraphy assessment were collected: 1) known risk factors for OSA (25, 26), such as smoking habit, BMI, presence of obesity and metabolic syndrome (MetS) according to IDF (27); 2) hormonal and metabolic parameters, such as GH, IGF-I, fasting glucose (FG), fasting insulin (FI), glycosylated hemoglobin (HbA1_c_), surrogate index of insulin resistance HOMA-IR, and lipid profile; 3) presence of acromegaly head modifications (macroglossia, prognathism, and malocclusion, 28) assessed by an experienced maxillofacial surgeon according to the Mallampati classification (29); 4) number of acromegaly complications (DM, hypertension, thyroid nodules, colon polyps).

Before polygraphy, all patients were administered with the validated sleep questionnaires, Epworth Sleepiness Scale (ESS) and STOP-BANG. The ESS questionnaire, used to measure daytime sleepiness, comprises 8 questions evaluating the likelihood of dozing in various situations over the past month, with scores ranging from 0 (never doze) to 3 (high chance), resulting in a total score between 0 and 24. An ESS score ≥10 was considered consistent with excessive daytime sleepiness (30). The STOP-Bang questionnaire comprises 8 dichotomous questions addressing factors such as snoring, daytime sleepiness, observed apnea episodes, hypertension, BMI, age, neck circumference, and gender (31). For each question, a “yes” response scores 1 point, while a “no” response scores 0 points, resulting in a total score that ranges from 0 to 8. Patients were classified as at risk for low, moderate or severe OSA with a STOP-Bang score of 0 to 2, 3 to 4, and 5 to 8, respectively.

### 2.4 Assays

All hormonal levels reported in this study have been analyzed according to international standards. IGF-I is expressed referred to the upper limit of normality (ULN) to eliminate the potential differences among the assays.

### 2.5 Statistical analysis

Data were analyzed using SPSS Software for Windows, version 30.0 (SPSS, Inc., Cary, NC package). Data are reported as Mean ± SD, unless otherwise specified for continuous variables and as percentage for categorical variables.

The Kolmogorov-Smirnov test was performed to define the normal distribution of considered variables, which resulted to have a not normal distribution. The comparison between the numerical data in different patient groups (patients with and without OSA) was made by Mann-Whitney test. The comparison between prevalence of different conditions was performed by *χ^2^* test, corrected by Fisher exact test when necessary. The extent of the risk was calculated and expressed as odds ratio (OR) (95% CI). The correlation study was performed by calculating Spearman’s correlation coefficients. Linear regression analysis was performed to identify the best predictor of OSA occurrence and severity in acromegaly. Significance was set at 5%.

## 3 Results

### 3.1 Obstructive sleep apnea

Complete data of acromegaly patients with and without OSA are shown in **Table 2**.

**Table 2:** Characteristics, prevalence and determinants of patients with No OSA and OSA (36 patients).

Variables	No OSA	OSA	p-value
	19 patients (52.8%)	17 patients (47.2%)	
*Men, (n, %)*	5 (26.3)	9 (52.9)	0.10
*Age at evaluation, (yrs)*	49.7 ± 11.1	59.1 ± 13.2	**0.008**
*Age at acromegaly diagnosis, (yrs)*	43.2 ± 10.6	51.1 ± 11.9	**0.02**
*Diagnostic delay before diagnosis, (yrs)*	5.0 ± 4.1	9.6 ± 4.6	**0.003**
*Smoking habit, (n, %)*	9 (47.4)	9 (52.9)	0.74
*GH, (ng/ml)*	2.9 ± 3.9	4.8 ± 7.2	0.52
*IGF-I, (ng/ml)*	279.0 ± 98.8	345.5 ± 269.2	0.82
*IGF x ULN*	1.0 ± 0.3	1.3 ± 0.8	0.44
*Fasting glucose, (mg/dl)*	96.3 ± 42.3	99.4 ± 17.4	0.10
*Fasting insulin, (UI/dl)*	10.3 ± 11.2	14.8 ± 11.9	0.13
*HOMA-IR*	3.3 ± 7.1	4 ± 3.5	**0.05**
*Insulin resistance, (n, %)*	4 (21%)	9 (52.9)	**0.05**
*HbA1c, (%)*	6.1 ± 1.6	6.0 ± 0.3	0.10
*Glucose profile impairment, (n, %)*	4 (21.0)	9 (52.9)	**0.05**
*DM, (n, %)*	3 (15.8)	6 (35.4)	0.18
*IFG, (n, %)*	1 (5.2)	3 (17.6)	0.29
*Total cholesterol, (mg/dl)*	179.9 ± 26.9	185.3 ± 45.1	0.90
*Triglycerides, (mg/dl)*	99.2 ± 41.3	115.5 ± 49.8	0.35
*HDL cholesterol, (mg/dl)*	57.1 ± 13.1	52.2 ± 11.8	0.32
*LDL cholesterol, (mg/dl)*	102.9 ± 20.7	110.0 ± 37.5	0.84
*Dyslipidemia, (n, %)*	7 (36.8)	12 (70.6)	**0.04**
*Hyperthension, (n, %)*	9 (47.4)	14 (82.3)	**0.03**
*BMI, (kg/m2)*	26.9 ± 5.9	30.1 ± 4.8	**0.03**
*Waist circumpherence, (cm)*	97.8 ± 18.3	104.4 ± 16.0	0.45
*Metabolic syndrome, (n, %)*	15 (20)	25 (55.5)	<0.001
*Acromegaly complications, (n, %)*	2.5 ± 1.8	3.9 ± 2.2	<0.001
*ESS score*	6.3 ± 3.9	6.3 ± 4.6	0.96
*STOP Bang score*	4.4 ± 1.4	2.5 ± 1.6	**0.001**

According to polygraphy findings, patients were classified in two groups, OSA (17 patients, 47.2%) and Not OSA (19 patients, 52.8%). Among patients of the first group, OSA was classified as mild in 11 (64.7%), moderate in 2 (11.8%), and severe in 4 (23.5%) patients. Patients with OSA were significantly older both at acromegaly diagnosis (p=0.02) and at polygraphy evaluation (p=0.008) as compared to those without OSA (**Figure 1**). Diagnostic delay before acromegaly diagnosis was significantly longer in patients with OSA as compared to those without (p=0.003, **Figure 1**). The prevalence of OSA in patients with a diagnostic delay > 6 years (median) was significantly higher than in those with a shorter diagnostic delay (68.4% *vs* 23.5%, p=0.007). Acromegaly diagnostic delay > 6 years was associated with 191% higher risk of OSA (RR = 2.91, 95% CI: 1.17-7.22). Conversely, acromegaly duration (i.e., the time between acromegaly diagnosis and OSA evaluation) was similar between the two groups (p=0.74, **Figure 1**).

**Figure 1:**
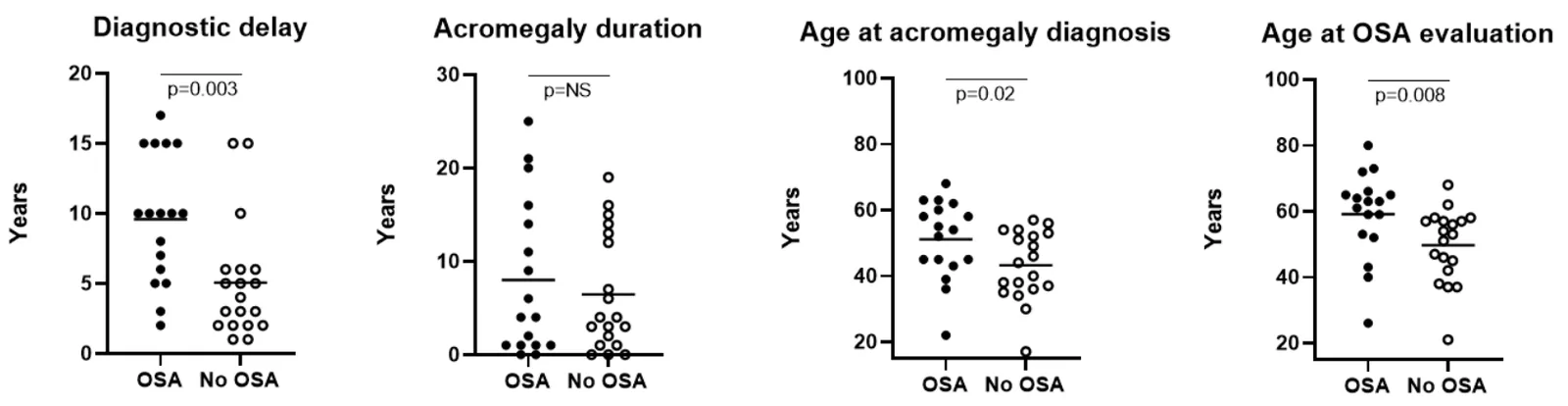
Differences in diagnostic delay, acromegaly duration, age at acromegaly diagnosis, and age at OSA evaluation between patients with and without OSA.

No significant difference in GH and IGF-I levels as well as in the prevalence of disease control was found between the two groups. BMI (p=0.03) and prevalence of obesity (p=0.05) were significantly higher in patients with OSA as compared to those without, whereas waist circumference was slightly but not significant higher in the former than in the latter (p=0.45). The prevalence of MetS was significantly higher in patients with OSA as compared to those without (p<0.001, **Figure 2**), as was the number of acromegaly complications (p<0.001). Although no significant difference in FG (p=0.11), FI (p=0.13), and HbA1c levels (p=0.11) was reported between patients with and without OSA, OSA patients exhibited a significantly higher HOMA-IR (p=0.05) and, consequently, a significantly higher prevalence of insulin-resistance (p=0.05, **Figure 2**) and of glucose profile abnormalities (p=0.05, **Figure 2**) as compared to patients without OSA. The prevalence of impaired fasting glucose (IFG) (p=0.29) and DM (p=0.18) were slightly higher in the former as compared to the latter. The prevalence of hypercholesterolemia was significantly higher among patients with OSA as compared to those without (70.6% *vs* 36.8%, p=0.04), thus resulting in a higher statin therapy requirement in the former as compared to the latter (41.2% *vs* 10.5%, p=0.04). The prevalence of hypertension was significantly higher among patients with OSA as compared to those without (p=0.03).

**Figure 2:**
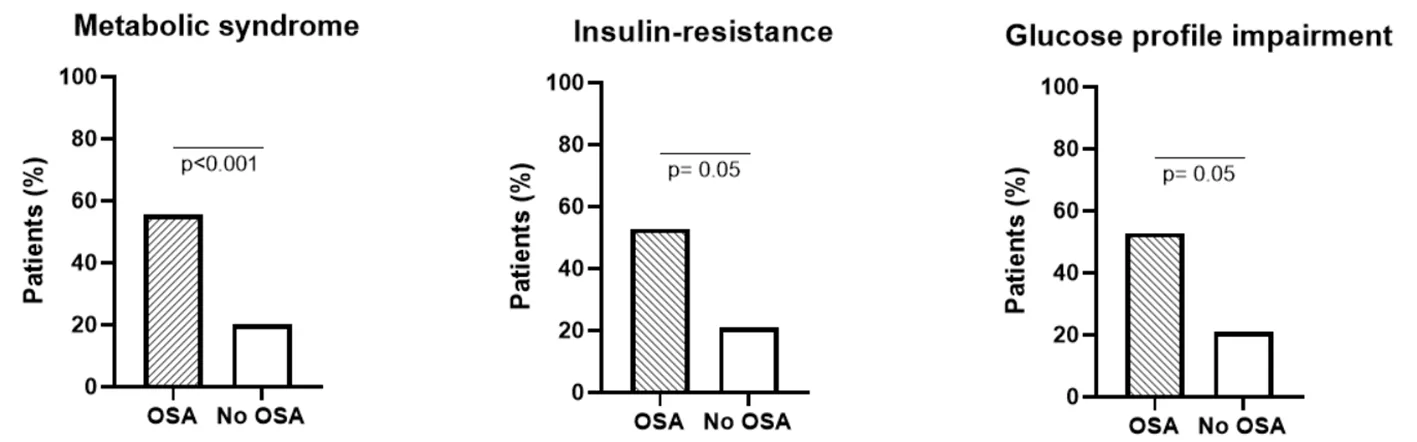
Prevalence of metabolic syndrome, insulin resistance, and glucose profile impairment between patients with and without OSA.

Among acromegaly head modifications, only malocclusion was slightly more frequent in patients with OSA as compared to those without (47.1% *vs* 21.1%, p=0.09), whereas the prevalence of macroglossia and prognathism resulted similar between the two groups. Mallampati classes 3 and 4 were slightly more frequent among patients with OSA as compared to those without (35.3% *vs* 14.3%, p=0.18).

Notably, no significant difference in the ESS score was found between patients with and without OSA (p=0.96), and the proportion of patients with an ESS score > 10, thus considered at high risk of daily sleepiness, was similar between the two groups (37.5% *vs* 25.0%, p=0.44).

Conversely, the STOP-Bang score was significantly higher in patients with OSA as compared to those without (p=0.001). Consistently, the proportion of patients with a STOP-Bang score of 0 to 2, thus considered at low risk for OSA, was significant lower (11.8% *vs* 47.3%, p=0.02) and the proportion of patients with a score of 5 to 8, thus considered at high risk of severe OSA, was significantly higher (52.9% *vs* 15.8%, p=0.02) in patients with OSA as compared to those without.

### 3.2 Positional obstructive sleep apnea

Complete data of acromegaly patients with and without pOSA are shown in **Table 3**.

**Table 3:** Characteristics, prevalence and determinants of patients with No pOSA and pOSA (36 patients).

Variables	No pOSA	pOSA	p-value
	25 patients (69.5%)	11 patients (30.5%)	
*Men, (n, %)*	9 (36.0)	5 (45.4)	0.59
*Age at evaluation, (yrs)*	52.7 ± 12.7	57.3 ± 13.4	0.11
*Age at acromegaly diagnosis, (yrs)*	45.8 ± 11.7	49.5 ± 12.1	0.24
*Diagnostic delay before diagnosis, (yrs)*	6.7 ± 5.1	8.2 ± 4.3	0.29
*Smoking habit, (n, %)*	14 (56.0)	4 (36.6)	0.28
*GH, (ng/ml)*	16.2 ± 27.3	8.7 ± 13.6	0.40
*IGF-I, (ng/ml)*	263.0 ± 107.2	394.0 ± 310.4	0.97
*IGF x ULN*	1.0 ± 0.4	1.4 ± 1.0	0.85
*Fasting glucose, (mg/dl)*	95.8 ± 37.1	102.6 ± 18.8	0.10
*Fasting insulin, (UI/dl)*	11.5 ± 11.7	14.8 ± 10.9	0.38
*HOMA-IR*	3.3 ± 6.3	4.3 ± 3.5	0.09
*Insulin resistance, (n, %)*	7 (28.0)	8 (72.7)	0.01
*HbA1c, (%)*	6.0 ± 1.4	6.1 ± 0.2	**0.04**
*Glucose profile impairment, (n, %)*	6 (24.0)	7 (6.3)	**0.05**
*DM, (n, %)*	4 (16.0)	6 (54.4)	**0.02**
*IFG, (n, %)*	2 (8.0)	1 (9.1)	**0.02**
*Total cholesterol, (mg/dl)*	174.9 ± 28.3	199.6 ± 44.7	0.13
*Triglycerides, (mg/dl)*	101.9 ± 41.1	118.4 ± 52.4	0.47
*HDL cholesterol, (mg/dl)*	55.7 ± 13.6	52.7 ± 9.6	0.58
*LDL cholesterol, (mg/dl)*	98.8 ± 22.4	122.9 ± 35.7	0.10
*Dyslipidemia, (n, %)*	12 (48)	7 (63.8)	0.52
*Hyperthension, (n, %)*	15 (60.0)	8 (72.3)	0.46
*BMI, (kg/m2)*	28.1 ± 6.3	29.1 ± 34	0.24
*Waist circumpherence, (cm)*	102.5 ± 19.4	98.7 ± 11.2	0.83
*ESS score*	6.3 ± 4.0	6.5 ± 4.5	0.89
*STOP Bang score*	4.0 ± 1.5	3.2 ± 1.9	0.22

According to polygraphy results, 11 patients (30.5%) had a positional OSA (pOSA). All patients with pOSA were affected by OSA. Patients with pOSA were slightly older at acromegaly diagnosis (p=0.24) and at polygraphy evaluation (p=0.11), and had a slightly longer diagnostic delay (p=0.29) as compared to those without.

No significant difference in GH and IGF-I levels, as well as in the prevalence of disease control was found between the two groups.

BMI was significantly higher in patients with pOSA as compared to those without (p=0.03), whereas obesity prevalence (p=0.80) and waist circumference (p=0.45) were not significant higher in the former as compared to the latter.

Patients with pOSA had significantly higher HbA1c levels (p=0.04) and slightly higher FG levels (p=0.10) as compared to those without pOSA. Consequently, the prevalence of all glucose profile abnormalities (p=0.02) and DM (p=0.02) was significantly higher in patients with pOSA as compared to those without, whereas a similar prevalence of IFG (p=0.91) was observed in the two groups. Despite similar FI levels (p=0.10), the prevalence of insulin-resistance expressed as a HOMA-IR >2.5 (72.7% *vs* 28%, p=0.01) was significantly higher in patients with pOSA as compared to those without.

No significant difference in prevalence of hypercholesterolemia (p=0.72) and of arterial hypertension (p=0.46) was found between the two groups.

Among acromegaly head modifications, only malocclusion was slightly more frequent in patients with pOSA as compared to those without (57.1% *vs* 28.6%, p=0.20), whereas macroglossia and prognathism prevalence resulted not significantly different between the two groups. The prevalence of Mallampati classes 3 and 4 was triple among patients with pOSA as compared to those without (45.4% vs 15.3%, p=0.06).

Notably, no significant difference in ESS (p=0.89) and STOP-Bang (p=0.22) scores was found between patients with and without pOSA.

### 3.3 Impact of acromegaly control on OSA prevalence and severity

Complete polygraphy data according to disease control are shown in **Table 4**.

**Table 4:** Analysis according to biochemical control of acromegaly.

	IGF-I < 1x ULN	IGF-I 1-1.3 ULN	IGF-I > 1.3 x ULN	p-value	p-value
	19 patients (52.8%)	9 patients (25%)	8 patients (22.2%)		IGF-I < 1x ULN *vs* IGF-I > 1.3 x ULN
*OSA, n (%)*	8 (42.1)	4 (44.4)	5 (62.8)	0.61	0.33
*pOSA, n (%)*	6 (31.6)	2 (22.2)	3 (37.5)	0.78	0.76
*AHI*	6.7 ± 8.3	9.5 ± 14.8	18.6 ± 20.1	0.16	0.06
*Number of hypopneas*	23.8 ± 31.2	40.2 ± 53.3	22.9 ± 28.9	0.93	0.77
*HI*	4.0 ± 6.3	5.2 ± 6.8	4.3 ± 6.3	0.93	0.73
*Number of apneas*	16.7 ± 16.2	33.0 ± 66.7	89.5 ± 133.5	0.06	**0.02**
*Number of obstructive apneas*	6.8 ± 8.7	29.2 ± 67.5	34.1 ± 34.1	0.16	**0.04**
*OAI*	0.9 ± 1.1	3.8 ± 8.6	7.6 ± 9.5	0.14	**0.03**
*Number of central apneas*	8.4 ± 11.2	2.5 ± 3.5	48.1 ± 111.6	0.54	0.98
*Number of mixed apneas*	1.5 ± 2.4	1.2 ± 1.6	7.2 ± 14.5	0.15	0.09
*CAI*	1.3 ± 1.8	0.3 ± 0.5	6.4 ± 15.2	0.51	0.86
*Mean duration apneas*	15.2 ± 5.4	16.0 ± 6.6	20.8 ± 9.2	0.44	0.24
*Mean duration hypopneas*	18.3 ± 4.9	16.7 ± 7.7	17.2 ± 7.4	0.86	0.51
*ODI*	7.0 ± 8.6	12.6 ± 17.8	18.4 ± 18.8	0.26	0.08
*T90*	3.3 ± 5.9	4.1 ± 6.0	21.9 ± 34.4	0.08	**0.03**
*Mean SpO2*	94.2 ± 1.7	93.9 ± 1.6	91.7 ± 4.4	0.19	0.07
*Mean duration desaturation*	45.5 ± 15.6	41.3 ± 15.3	36.6 ± 8.1	0.28	0.16
*Nadir SpO2*	84.6 ± 5.4	83.0 ±6.1	77.5 ± 8.8	0.08	**0.02**
*ESS score*	6.3 ± 44	5.9 ± 4.1	7.0 ± 4.4	0.73	0.52

Prevalence of OSA was 42.1% in controlled, 44.4% in partly controlled, and 62.8% in uncontrolled patients (p=0.61), whereas prevalence of pOSA was 31.6% in controlled, 22.2% in partly controlled, and 37.5% in uncontrolled patients (p=0.78).

Apart a slight difference in the number of apneas (p=0.06), T90 (p=0.08), and nadir SpO2 (p=0.08), no significant difference among the three groups was found. Conversely, several significant divergences were found when comparing patients with fully controlled and uncontrolled disease. Although AHI was only slightly higher (p=0.06) in uncontrolled patients as compared to those with full disease control, the number of apneas (p=0.02) and obstructive apneas (p=0.04) were significantly higher in the former as compared to the latter (**Figure 3**). The number of central apneas was not significantly different between these two groups, whereas the number of mixed apneas (p=0.09) was slightly higher in uncontrolled as compared to fully controlled patients. OAI was significantly higher in uncontrolled patients as compared to those with full disease control (p=0.03, **Figure 4**). T90 was significantly higher (p=0.03) and nadir SpO2 significantly lower in uncontrolled patients as compared to those fully controlled (p=0.02, **Figure 4**). ODI was slightly higher (p=0.08) and mean SpO2 lower (p=0.07) in uncontrolled patients as compared to those fully controlled.

**Figure 3:**
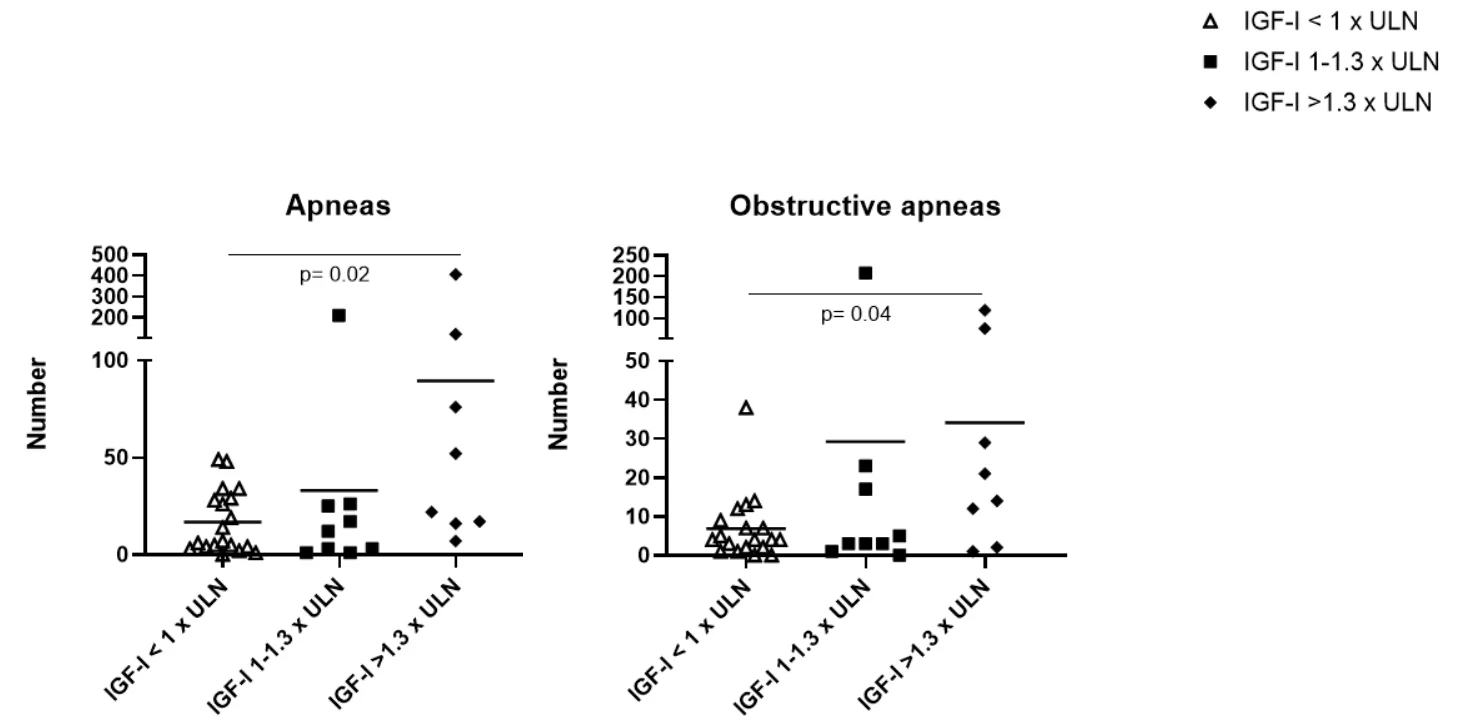
Differences in number of sleep apneas and obstructive sleep apneas according to biochemical control of acromegaly.

**Figure 4:**
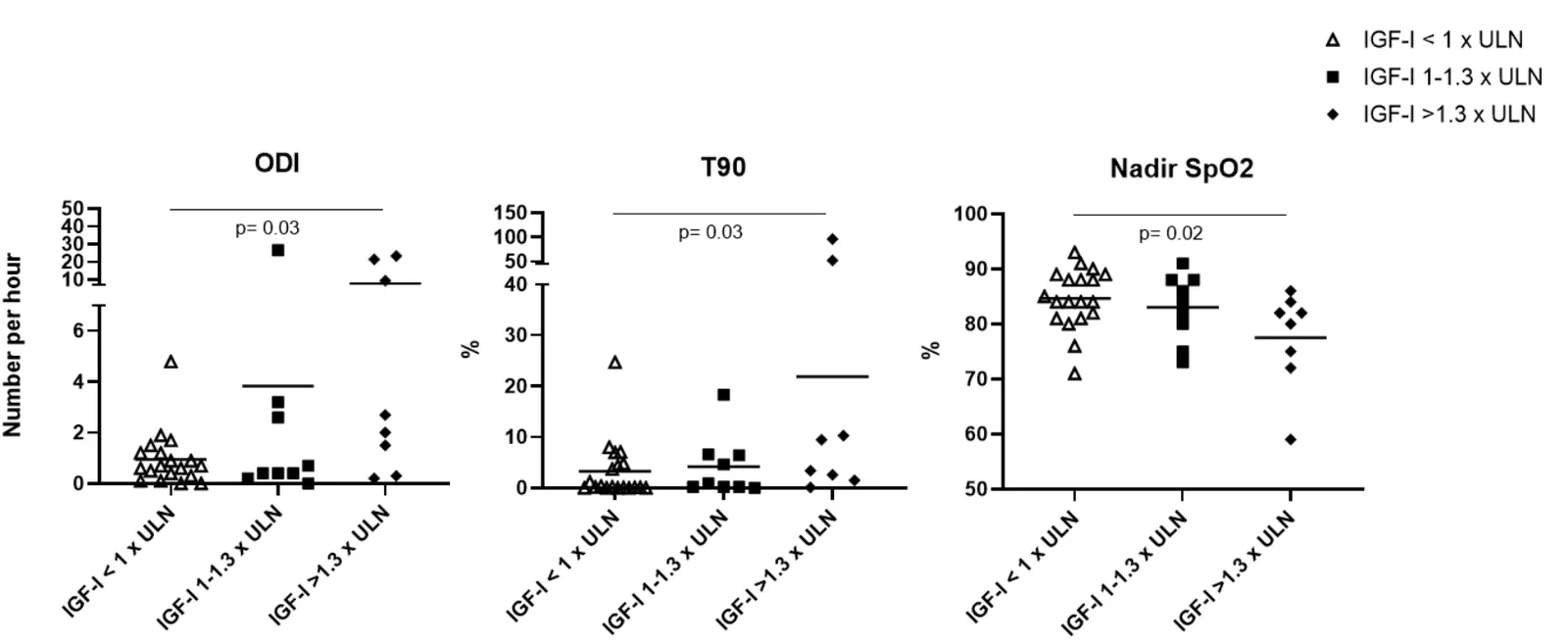
Differences in ODI, T90, and nadir SpO2 according to IGF-I values according to biochemical control of acromegaly.

ESS (p=0.73) and STOP-Bang (p=0.30) scores were not significantly different among groups (**Table 4**).

Macroglossia was slightly more common in uncontrolled patients as compared to those fully controlled (66.7% *vs* 36.4%, p=0.23) as was for Mallampati classes 3 and 4 (37.5% *vs* 21%, p=0.37).

### 3.4 Evaluation of determinants of polygraphy indices

AHI score was significantly correlated with diagnostic delay (r=0.395, p=0.02), but not with age at acromegaly diagnosis (r=0.188, p=0.28) and age at polygraphy evaluation (r=0.311, p=0.07), independently on BMI. At regression analysis, diagnostic delay was the best predictor of AHI score (t=2.103, p=0.04).

HI score significantly correlated with BMI (r=0.352, p=0.03), but not with diagnostic delay (r=0.219, p=0.21) and age at acromegaly diagnosis (r=0.06, p=0.707). Glucose levels (r=0.285, p=0.09) and HOMA-IR (r=0.318, p=0.07) only slightly but not significantly correlated with HI score.

The desaturation index ODI significantly correlated with diagnostic delay (r=0.442, p=0.008), age at polygraphy evaluation (r=0.401, p=0.02), and BMI (r=0.404, p=0.01), but not with age at acromegaly diagnosis (r=0.299, p=0.08). At regression analysis, diagnostic delay was the best predictor of ODI score (t=2.173, p=0.04), independently on BMI.

The percentage of peripheral Oxygen Saturation SpO2 inversely correlated with the diagnostic delay (r=-0.439, p=0.008) and age at lung function evaluation (r=-0.492, p=0.03), but not with age at acromegaly diagnosis (r=-0.316, p=0.06), independently on BMI. At regression analysis, age at polygraphy evaluation was the best predictor of SpO2 (t=2.79, p=0.009).

The time spent with a SpO2 < 90% inversely correlated with BMI (r=-0.386, p=0.02), but not with diagnostic delay (r=0.143, p=0.41) and age at acromegaly diagnosis (r=-0.22, p=0.89).

STOP-Bang, but not ESS, score correlated with age at polygraphy evaluation (r=0.455, p=0.006), and BMI (r=0.462, p=0.005), but not with diagnostic delay (r=0.295, p=0.08). At regression analysis, BMI was the best predictor of STOP-Bang score (t=2.56, p=0.01). STOP-Bang score correlated with several polygraphy indices, including AHI (r=0.613, p<0.001), HI (r=0.563, p<0.001), ODI (r=0.577, p<0.001), T90 (r=0.469, p<0.001), mean SpO2 (r= -0.549, p<0.001), and nadir SpO2 (r= -0.421, p=0.01).

## 4 Discussion

The current is the first study demonstrating the impact of diagnostic delay and biochemical control on OSA prevalence and severity in a cohort of acromegaly patients. Additionally, the present is the first study investigating the prevalence and characteristics of p(OSA) and the potential role of the STOP-Bang questionnaire as a screening tool for OSA in acromegaly patients.

According to polygraphy results, approximately half of patients of the current cohort exhibited OSA; these data are in line with previous results, reporting OSA in 43.7- 97% of different acromegaly patients series (4, 6, 7, 9–14). To note, across the studies the prevalence and the severity of OSA have been assessed using different methods and tools, and a notable heterogeneity in disease status has been included, leading to the conclusion that available results about the prevalence of OSA are not fully comparable in the literature. Noteworthy, overall OSA prevalence in newly diagnosed patients (75.0%, 4, 7, 10, 13, 14) appears to be similar to that reported in studies investigating sleep disorders in acromegaly patients regardless of disease status, thus including both controlled and uncontrolled patients (69.4%, 6, 11, current study). However, two-thirds of the patients included in the current investigation were controlled, either fully (52.8%) or partly (25%), whereas only 22.2% were persistently uncontrolled at the time of lung function evaluation, consistently with a previous study (6) reporting a similar prevalence of OSA in a mixed population of controlled (63.3%) and uncontrolled (36.7%) patients. Altogether, these findings raise the hypothesis that disease status might influence overall prevalence of OSA in the current series.

In our study, patients with OSA were significantly older both at acromegaly diagnosis and at polygraphy evaluation as compared to those without, underlining the primary role of patients’ age on respiratory impairment. Patient’s age has commonly been hypothesized as a determinant of OSA in patients with acromegaly, but with inconsistent results, since some studies confirmed this hypothesis (4, 9, 10), whereas others reported no significant difference in age between patients with and without OSA (6, 11). The current study not only demonstrated the role of patient’s age in respiratory impairment, but also first reported that age at polygraphy was the best predictor of oxygen desaturation (SpO2), being older patients at higher risk of lower peripheral oxygen saturation. Based on these findings, a special attention should be paid at polygraphy in elderly patients with acromegaly, who are at higher risk of OSA and desaturation as compared to younger acromegaly patients.

For the first time, the role of the exposure length to GH and IGF-I excess on respiratory impairment has been investigated in the current study, and diagnostic delay has been demonstrated to be the main determinant of OSA prevalence and severity. Indeed, patients with a longer diagnostic delay almost triple risk of OSA development as compared to those with a shorter diagnostic delay. Moreover, the correlation between the length of diagnostic delay and OSA severity appears to suggest a potential direct impact of diagnostic delay on the number of apneas, hypopneas, and desaturation episodes per hour. This is a novel and relevant finding in the context of OSA in acromegaly patients and might be linked to the higher prevalence of comorbidities in elderly acromegalic subjects. Indeed, the number of comorbidities has been demonstrated to be significantly higher in older patients with a longer diagnostic delay, particularly for those with a diagnostic delay >5 years, as compared to those early diagnosed with acromegaly (32). Indeed, diagnostic delay is known to be one of the main determinants for the development of several chronic complications of acromegaly such as colon polyps (33), cardiovascular disease (34), and impaired nervous and vascular retinal structure (35). It can be hypothesized that, similarly to organs rearrangements demonstrated in other body districts, during the years of exposure to unrecognized GH and IGF-I excess, several anatomical changes of the upper respiratory tract could occur, such as thickening of tongue base, pharyngeal wall, and cartilages which could lead to the reduction of the cross-sectional area of the airway (4, 6, 7). It is plausible to assume that these anatomical changes of the upper airways could affect nocturnal breathing, and, although they may improve with disease control especially due to the deflation of soft tissues, they might persist even after a long time from diagnosis, particularly in the more severe cases of remodeling of cartilage and bone structures. Consistently, malocclusion and Mallampati classes 3 and 4 were reported more frequently among patients with OSA as compared to those without in this study. These head modifications require a long time to develop and are not reversible (2), so it can be postulated that a shorter diagnostic delay might prevent the development of these anatomical changes and, therefore, of OSA, whereas a longer diagnostic delay could foster these head modifications with consequent OSA progression. In this light, an early diagnosis of acromegaly should be encouraged to help in decreasing the prevalence and severity of OSA.

Besides the importance of early diagnosis, also prompt acromegaly control is of crucial importance in OSA management. According to the results of the current study, disease control might mainly influence the severity of OSA rather than the prevalence. In fact, no significant difference has emerged in the prevalence of OSA according to acromegaly control, whereas a significant difference was found in OSA severity when comparing patients with full disease control and those uncontrolled. Based on these findings, disease severity (i.e., IGF-I levels) could be hypothesized to act mainly on upper airways soft tissues edema and thickening, for example by determining macroglossia, which was indeed more common in uncontrolled patients, as was for Mallampati classes 3 and 4 in the current study. Overall, according to the results of the current study, we can presume that the exposure to excessive levels of GH and IGF-I for a long time leads to head anatomical changes, thus determining OSA, whereas full disease control might lead to a deflation of the tongue and soft tissues of the respiratory airways allowing the improvement in OSA severity in those who develop OSA before acromegaly diagnosis. These data are in line with a previous study (13), reporting the persistence of OSA despite disease control, particularly in those with severe OSA. Therefore, diagnostic delay exerts a greater impact than disease status on OSA prevalence and severity.

In this study, OSA was linked to metabolic syndrome, insulin-resistance, and glucose profile impairment. This is a novel finding for the acromegaly population, albeit widely explored in general population (36). Indeed, OSA is a known risk factor for DM independently on age, sex, and BMI in the general population (36). A recent study reported that OSA severity, evaluated using AHI index, was significantly associated to patients’ weight, neck and waist circumpherence in general population (37). The impairment of glucose metabolism is assumed to be ascribable to sympathetic activation, systemic inflammation, and inflammatory cytokines secretion, together leading to counter-regulatory hormones secretion, fatty acids production, and β-cell dysfunction (38), and consequently to a higher risk of cardiometabolic disease. Similarly, in acromegaly patients the impairment in glucose metabolism has been linked to an increased cardiovascular morbidity and mortality (16). Indeed, the coexistence of DM and cardiovascular disease, such as arterial hypertension, induces cardiac dysfunction (39). The association of acromegaly and IGT or DM has been reported to promote a lower left ventricular ejection fraction and maximal early to late diastolic flow velocity ratio, thus triggering diastolic dysfunction (39), along with a thickening of the left ventricular posterior wall, thus eliciting the peculiar cardiac hypertrophy (39), which together contribute to the increased cardiovascular mortality reported in acromegaly patients (3).

Altogether, these considerations lead to the conclusion that in acromegaly an early screening for respiratory complications, and particularly OSA, should be encouraged to help endocrinologists in predicting associated cardiometabolic disease and mortality, and polygraphy should be offered to all acromegaly patients at diagnosis and thereafter. In this light, the availability of easy screening tools, such as sleepiness self-reporting questionnaires, might assist in the prompt identification of patients at greater risk. Nowadays, current guidelines recommend the use of ESS score to identify patients at risk of OSA (5). However, in the current study, ESS failed to detect all patients at OSA risk, as no significant difference in ESS score was found between patients with and without OSA. In the current experience, the STOP-Bang questionnaire scores, commonly used for the assessment of sleep disorders (31), was able to discriminate acromegaly patients with and without OSA, thus supporting its potential future use in clinical settings.

This study presents several limitations, which however do not affect the importance of the results obtained. This is a single-center study on a relatively small patient population. Diagnostic delay has been calculated based on patients interviews and photographs, which might result in a biased estimation of this parameter. However, the global study methodology, the appropriateness of the study population totally consistent with the power sample analysis, and the use of a diagnostic delay assessment methodology widely consolidated in previous studies make the results of this study able to suggest the role of diagnostic delay as a potential determinant of OSA prevalence and severity.

## 5 Conclusions

In conclusion, the results of the current study confirmed that OSA represent a frequent comorbidity in acromegaly, affecting almost half of patients. Diagnostic delay and patients ‘age, rather than disease status, appear to predict the development of OSA and influence its severity. In turn, OSA is strongly linked to cardiometabolic complications, because OSA is associated with insulin resistance, metabolic syndrome, arterial hypertension and hypercholesterolemia in acromegaly patients, as in the general population. In this light, an early identification of patients at high OSA risk may contribute to decrease the risk of cardiometabolic impairment and, potentially, to reduce cardiovascular mortality. Future studies will help in elucidating the role and the burden of OSA in acromegaly permanent disability and mortality.

## Data Availability

The raw data supporting the conclusions of this article will be made available by the authors, without undue reservation.
